# *In vivo* volumetric analysis of retinal vascular hemodynamics in mice with spatio-temporal optical coherence tomography

**DOI:** 10.1117/1.NPh.11.4.045003

**Published:** 2024-10-08

**Authors:** Piotr Węgrzyn, Wiktor Kulesza, Maciej Wielgo, Sławomir Tomczewski, Anna Galińska, Bartłomiej Bałamut, Katarzyna Kordecka, Onur Cetinkaya, Andrzej Foik, Robert J. Zawadzki, Dawid Borycki, Maciej Wojtkowski, Andrea Curatolo

**Affiliations:** a International Centre for Translational Eye Research, Warsaw, Poland; b Polish Academy of Sciences, Institute of Physical Chemistry, Warsaw, Poland; c University of Warsaw, Faculty of Physics, Warsaw, Poland; d University of California Davis, Department of Ophthalmology and Vision Science, Sacramento, California, United States; e Nicolaus Copernicus University, Faculty of Physics, Astronomy and Informatics, Toruń, Poland; f Politecnico di Milano, Department of Physics, Milan, Italy

**Keywords:** hemodynamics, spatio-temporal optical coherence tomography, retinal imaging, choroid, mouse, neurovascular coupling

## Abstract

**Significance:**

Microcirculation and neurovascular coupling are important parameters to study in neurological and neuro-ophthalmic conditions. As the retina shares many similarities with the cerebral cortex and is optically accessible, a special focus is directed to assessing the chorioretinal structure, microvasculature, and hemodynamics of mice, a vital animal model for vision and neuroscience research.

**Aim:**

We aim to introduce an optical imaging tool enabling *in vivo* volumetric mouse retinal monitoring of vascular hemodynamics with high temporal resolution.

**Approach:**

We translated the spatio-temporal optical coherence tomography (STOC-T) technique into the field of small animal imaging by designing a new optical system that could compensate for the mouse eye refractive error. We also developed post-processing algorithms, notably for the assessment of (i) localized hemodynamics from the analysis of pulse wave–induced Doppler artifact modulation and (ii) retinal tissue displacement from phase-sensitive measurements.

**Results:**

We acquired high-quality, *in vivo* volumetric mouse retina images at a rate of 113 Hz over a lateral field of view of ∼500  μm. We presented high-resolution *en face* images of the retinal and choroidal structure and microvasculature from various layers, after digital aberration correction. We were able to measure the pulse wave velocity in capillaries of the outer plexiform layer with a mean speed of 0.35 mm/s and identified venous and arterial pulsation frequency and phase delay. We quantified the modulation amplitudes of tissue displacement near major vessels (with peaks of 150 nm), potentially carrying information about the biomechanical properties of the retinal layers involved. Last, we identified the delays between retinal displacements due to the passing of venous and arterial pulse waves.

**Conclusions:**

The developed STOC-T system provides insights into the hemodynamics of the mouse retina and choroid that could be beneficial in the study of neurovascular coupling and vasculature and flow speed anomalies in neurological and neuro-ophthalmic conditions.

## 1 Introduction

The retina and optic nerve are complex neural tissues originating from the diencephalon and connected to the central nervous system (CNS), where visual processing takes place in the visual cortex. The retina shares many similarities with the cerebral cortex, such as ganglion cells with the typical morphology of CNS neurons. It has been shown that many CNS diseases, including multiple sclerosis, Alzheimer’s disease, and Parkinson’s disease, can also be detected on the retina level.^1^ Given that around 80% of external information is processed through visual perception^2^ and with retina-related blindness being a significant global health issue,^3^ understanding retinal structure and function, vascular hemodynamics, and neurovascular coupling is of paramount importance.^4^

For example, retinal ganglion cell axons are nonmyelinated within the retina. Thus, the retinal nerve fiber layer (NFL) is an optimal structure for visualizing the process of neurodegeneration, neuro-protection, and neuro-repair.^5^ Moreover, despite extensive studies of alterations to blood circulation in the CNS and optic nerve in neurological and neuro-ophthalmological diseases, it is still unclear whether the vascular alterations are secondary to neuronal loss from decreased metabolic demand and neurovascular coupling or a pathogenic step preceding neurodegeneration. Retinal vascular features, including vascular attenuation, tortuosity, and foveal avascular zone alterations, as well as structural changes in retinal and choroidal layer thickness, have received increasing attention in the setting of neurological and neuro-ophthalmological diseases.^6^

The retina is conveniently located at the imaging plane of the optical system of the eye, thus providing not only a “window” to a sensory peripheral of the brain but also the opportunity for non-invasive observation of alterations associated with neurodegenerative conditions and changes in neural tissue vasculature.^7^

Recent advancements in retinal imaging are enabling the exploitation of this potential.^4^ Scanning laser ophthalmoscopy (SLO)^8^ and optical coherence tomography (OCT)^9^ are two complementary *in vivo* retinal imaging modalities that have often been combined with adaptive optics (AO) to successfully image the photoreceptor mosaic, the retinal microvasculature,^10^^–^^12^ and the highly transparent ganglion cell somas in the human inner retina^13^^,^^14^ and its temporal dynamics.^15^

Retinal blood flow can be monitored quantitatively *in vivo* through laser speckle flowgraphy,^16^ a technique that produces angiographic contrast from speckle variance, allowing for full-field blood flow measurements (in arbitrary units) with a high temporal resolution. Alternatively, laser Doppler flowmetry^17^ can measure blood flow and mean velocity in relative units, and with its full-field extension, laser Doppler holography (LDH)^18^ can estimate retinal pulsatile flow over a lateral field of view (FOV) with a millisecond resolution. The aforementioned techniques lack depth sectioning ability, making the influence of choroidal flow unclear. However, it would be beneficial to analyze choroidal hemodynamics^19^^,^^20^ separately from that of the inner retina and at high temporal resolution.

OCT angiography (OCT-A)^21^^,^^22^ and Doppler OCT^23^ provide depth-sectioned hemodynamic information; however, these approaches have been most commonly implemented with flying-spot OCT systems, where the limited volumetric imaging rates restrict the temporal resolution needed to monitor the blood flow during a cardiac cycle within the vascular network in 3D. Fourier-domain full-field optical coherence tomography (FD-FF-OCT) overcomes the acquisition speed issue, and it has been employed for functional neuronal analysis^24^^,^^25^ and hemodynamic analysis, including pulse wave propagation in human retinal vessels^26^ and in combination with LDH.^27^ Choroidal vessel visibility, though, remains limited in structural FD-FF-OCT images due to coherent crosstalk noise that limits imaging of deeper layers located in scattering media. Spatio-temporal optical coherence tomography (STOC-T), a variant of FD-FF-OCT^28^^,^^29^ suppressing crosstalk via spatio-temporal optical phase manipulation,^30^ has demonstrated an enhanced ability to image the full thickness of the chorio-retinal complex,^31^ and thanks to its high phase stability and ultrafast volumetric imaging rate, it has been successfully employed in functional imaging of photoreceptor response in humans.^32^^,^^33^

The application of the aforementioned optical retinal imaging techniques in mice^34^ is particularly important as rodents serve as a primary animal model for exploring not only fundamental aspects of vision science and ophthalmic research,^35^^–^^37^ including investigations into phototransduction mechanisms,^38^^,^^39^ but also broader applications in the field of general neuroscience.^7^ Various groups studied neurovascular coupling in rodents subject to flicker stimulation.^40^^,^^41^ Inner retinal blood flow has been monitored with LDH in rodents at a temporal resolution of ∼6.5  ms.^42^ Ultrahigh-resolution images of the mouse retina have been acquired with visible light OCT^43^ and AO-SLO.^44^ Zhang et al.^45^^,^^46^ demonstrated *in vivo* cellular resolution neuronal and vascular retinal imaging in mice without AO and, more recently, with AO.^47^ A sensorless AO approach has also provided volumetric cellular imaging of microglia throughout the inner retina.^48^

Although these advances show tremendous progress toward elucidating the relationship between the neuronal and vascular retinal tissues in health and disease models, the use of STOC-T in mouse retinal imaging would hold promise to complement the aforementioned techniques by providing high-resolution imaging down to the choriocapillaris,^31^ several hemodynamic signals at millisecond temporal resolution for angiography and neurovascular coupling, and functional information within the same system. In fact, owing to its high phase stability within a single volume, digital aberration correction (DAC) can be applied^49^^,^^50^ to approach the resolution obtained with AO, whereas phase stability over consecutive volumes would allow retinal functional and hemodynamic signal measurements, including quantitative information about the retinal tissue expansion from blood vessel pulsation, useful in the study of neuro-vascular coupling and vascular and optic nerve head biomechanics.^51^^,^^52^ Meanwhile, STOC-T structural 3D images of the retina would allow the monitoring of dynamic changes at a temporal resolution of a few milliseconds.

This work describes a STOC-T system designed for ultrafast volumetric mouse retinal imaging that can be controlled to focus on a specific retinal or choroidal vascular bed in the mouse eye, using a calibrated fundus imaging system. The STOC-T image processing with numerical dispersion compensation, DAC, and volume co-registration with sub-pixel precision attains high-spatial and temporal resolution retinal and choroidal images. A simple STOC-T amplitude-only processing allows the extraction of pulse traces from different retinal and choroidal vessels, the estimate of pulse wave velocities in capillaries, and the evaluation of arterial and venous pulse delay. On the other hand, phase-sensitive processing enables retrieval of other hemodynamic signals, including tissue displacement caused by blood vessel pulsation.

## 2 Methodology

**Fig. 1:**
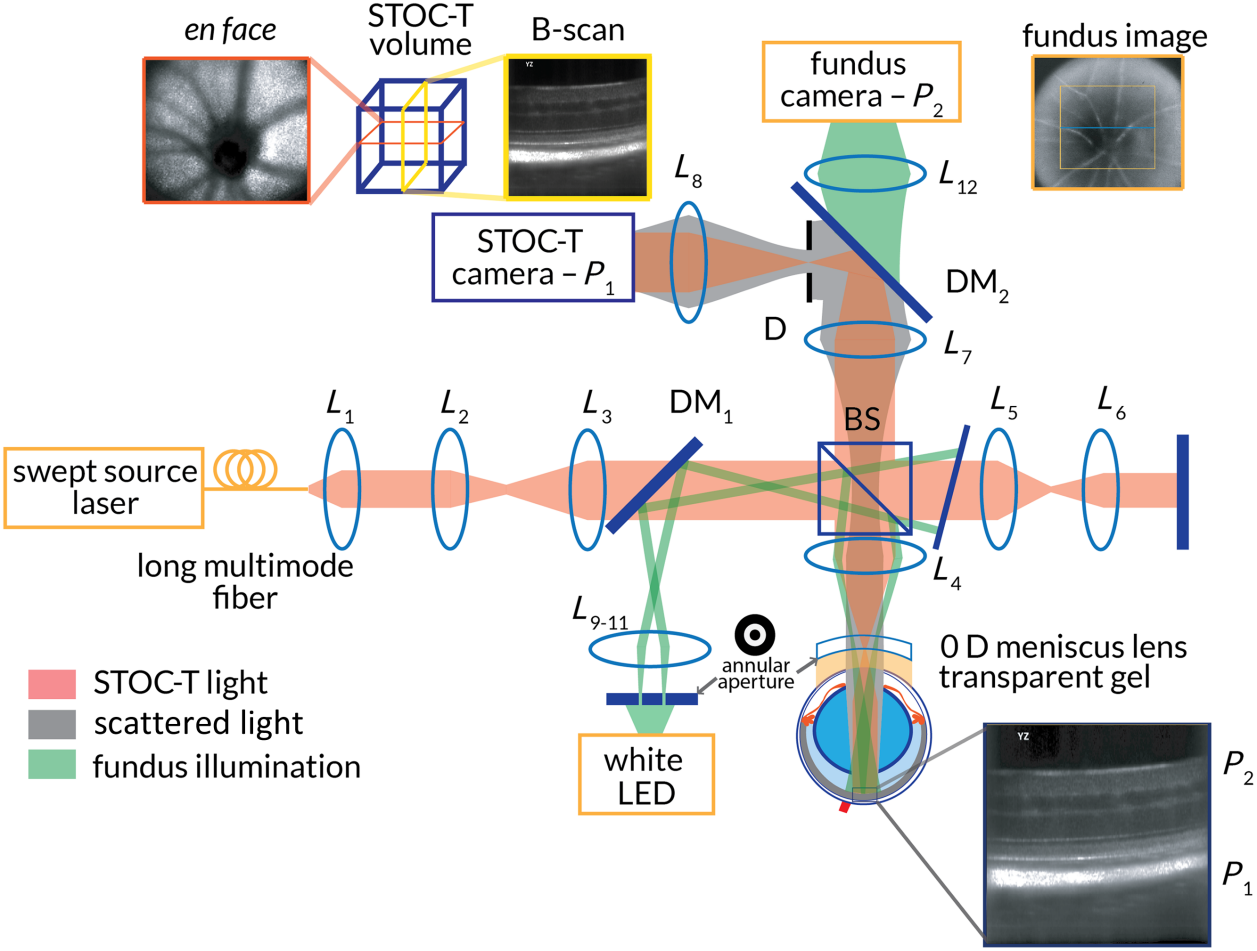
Schematic of the STOC-T experimental setup for mouse retinal imaging and fundus imaging system to support the alignment of the mouse for proper focusing on the retinal layer of interest. Bringing the retinal NFL (labeled $P_{2}$) into focus on the fundus camera facilitates the focusing of the STOC-T image on the photoreceptor layer in the retina (labelled $P_{1}$), after a calibration procedure (described in the Appendix B) sets the offset between the focal planes of two imaging systems.

The proposed high-speed volumetric mouse retinal imaging system and its application to vascular hemodynamics involve the development of a custom optical setup and several data processing steps described in the Secs. 2.1–2.7. The optical setup, presented in Fig. 1, is based on the following features: a STOC-T imaging system comprising a Michelson interferometer with a sample interface that can compensate for the mouse eye refractive error^53^ and a white-light fundus imaging system^54^ for aiding the mouse’s eye alignment.

### 2.1 Mouse Retinal STOC-T Imaging System

The STOC-T imaging technique combines FD-FF-OCT with control over the spatial coherence characteristics of the employed near-infrared (NIR) light, with the added benefit of reducing the influence of coherent crosstalk noise, and therefore accessing deeper layers in the eye.^31^ Spatial coherence reduction is achieved by delivering NIR light from a swept-source laser to the optical interferometer via a long multimode (MM) fiber, creating many temporally incoherent spatial modes (TEMs). At the detector, the TEMs cause an incoherent summation of interference signals adding constructively when the angular spectrum of the TEM scattered by the sample matches that of the corresponding TEM reflected from the reference mirror. Thus, the interference of TEMs that are strongly distorted by scattering has a very low contrast, reducing the contribution of the crosstalk noise to the interferometric signal.^55^

More specifically, NIR light from the swept-source laser (BS-840-2-HP, Superlum, Co. Cork, Ireland) is delivered to the system via 300 m of an MM fiber with a 50  μm core diameter (FG050LGA, Thorlabs, Newton, New Jersey, United States).^55^^,^^56^ Light is then collimated by an aspheric lens collimator $L_{1}$ (APC22-780, Thorlabs), expanded by a telescope comprising two achromatic doublet lenses $L_{2}$ (f=30  mm) and $L_{3}$ (f=75  mm), and split into the reference arm and the sample arm by a beam splitter. The optical components in the sample arm comprise an achromatic doublet lens $L_{4}$ (f=50  mm) and a zero-diopter (0 D) meniscus lens (Cantor & Nissel, Brackley, United Kingdom) with lubricant gel (Vidisic, Bausch & Lomb, Rochester, New York, United States) as the sample interface. The mouse eye is brought in contact with the gel, and the gel thickness is adjusted by manipulating the mouse position with respect to the meniscus lens. Lens $L_{4}$ focuses the image of the MM fiber tip near the front focal plane of the mouse eye. Therefore, the mouse retina is illuminated by a low-divergence beam creating an illumination area on the retina with a diameter of ∼450  μm. NIR light backscattered by the retina propagates back through the eye, $L_{4}$, and the beam splitter to form an image of the retina in an intermediate conjugate plane relayed to the sensor of a high-speed camera (Nova S16, Photron, Tokyo, Japan) by a 1:1 telescope comprising two 2” achromatic doublet lenses $L_{7}$ and $L_{8}$ (f=75  mm) in the detection path. An iris is introduced in a conjugate plane of the mouse pupil behind $L_{7}$ to control the aperture of the system and, therefore, the lateral resolution and depth of field (DOF).

Light in the reference arm gets reflected by a silver mirror after propagating through an achromatic doublet $L_{5}$ (f=50  mm) and a small plano-convex lens $L_{6}$ (f=2  mm, 2.5 mm aperture) that mimics the focal length of the mouse eye to provide a similar field curvature at the STOC-T camera as the one from the sample arm. A neutral density filter is used to optimize the reference arm power within the STOC-T camera dynamic range to maximize the sensitivity.

The STOC-T system performs a sweep of the laser from 803 to 878 nm linearly in wavenumber units, whereas the ultrafast camera records 512 images with a resolution of 512 by 448 pixels at a frame rate of 60,000 fps. This results in a volumetric scan rate of ∼113  Hz, meaning that hemodynamic signals within the captured volume can be analyzed every 8.9 ms.

Additional specifications of the mouse retinal STOC-T system are reported in Table 1.

**Table 1:** Mouse retinal volumetric imaging system specifications

Specification	STOC-T system	Fundus system
Central wavelength/bandwidth/instantaneous linewidth	840 nm/75 nm/0.12 nm	600 nm/137.5 nm/N/A
Axial resolution in air (theoretical/measured)	4.2 μm/5.0 μm	N/A
Lateral resolution (measured in mouse eye phantom—see Appendix A)	1.74 μm	1.23 μm
Illumination diameter (on mouse eye phantom retina)	∼450 μm	∼1.2 mm
FOV (camera-limited)	∼530 μm	∼945 μm
Optical power at the cornea	9.1 mW	100 μW
Sensitivity from a single volume	78.01 dB	N/A
Volume acquisition rate	112.8 Hz	N/A

### 2.2 Mouse Eye Fundus Imaging System

The mouse retinal volumetric imaging system includes a mouse eye fundus imaging system. This consists of a white light LED source (MWWHL4, Thorlabs) followed by a telescope, comprising two achromatic doublet lenses $L_{9}$ (f=30  mm) and $L_{10}$ (f=40  mm), forming a conjugate image at a position where we introduced an amplitude mask, shaping the source as an annulus. The annulus image is relayed by the achromatic doublet lens $L_{11}$ (f=75  mm), via a dichroic mirror ${\mathrm{DM}}_{1}$ (DMLP650, Thorlabs), and lens $L_{4}$ onto the meniscus lens front surface. This produces two effects: it avoids specular reflection from the meniscus lens apex, which would severely degrade the contrast of the fundus image, and it goes on to illuminate the mouse retina in a Kohler illumination scheme. White light backscattered from the mouse eye fundus propagates through the mouse eye, gel and meniscus lens, lens $L_{4}$, the beam splitter, lens $L_{7}$, the dichroic mirror ${\mathrm{DM}}_{2}$ (#69-207, Edmund Optics, Barrington, New Jersey, United States), and the achromatic doublet lens $L_{12}$ (f=40  mm) in the detection path to focus on the fundus camera (ac1300-200um, Basler, Highland, Illinois, United States). The fundus imaging system specifications are reported in Table 1. Fundus image processing involves background subtraction from the raw fundus images to further reduce stray reflections that reduce the image contrast. The background image was acquired with the mouse eye separated from the meniscus lens by a substantial amount of gel, before bringing the fundus into focus by moving the mouse closer to the meniscus lens.

### 2.3 Imaging Mouse Eyes *In Vivo*

The study described in the paper was approved by the First Local Ethics Committee for Animal Experiments in Warsaw regarding all protocols for mouse rearing and handling (resolution no 1401P3/2022). Albino (balb/c) and wild-type (C57/BL6) mice were obtained from the Nencki Institute of the Polish Academy of Sciences, in Warsaw, Poland. Transparent lubricant gel (Vidisic, Bausch & Lomb) was applied to the mouse eye to prevent cold cataracts and to maintain the hydration and transparency of the eye anterior segment. During the experimental procedure, mice were anesthetized with 1.5% isoflurane (Vetflurane 100 mg/g, Virbanc, Carros, France), delivered in $O_{2}$, and maintained on an adjustable platform equipped with a heating blanket (additional details in the Appendix C). Mouse pupils were dilated with medical-grade tropicamide (Tropicamidum WZF 1%, Polfa Warszawa, Warsaw, Poland) and phenylephrine (Neosynephrin-POS 10%, Ursapharm, Saarbrucken, Germany).

In terms of mouse eye safety from photothermal damage, there are no standards for maximum permissible exposure (MPE), and studies on rat retinas^57^ have shown that it is not easy to translate the human retina MPE to rodents by simply scaling based on numerical aperture. However, we carefully evaluated that no damage was visible in the mouse retina after our measurements and confirmed its retained functionality with electrophysiology tests.

### 2.4 Mouse Refractive Error Compensation

Mouse eyes can present refractive errors, and it is reported that mouse eyes are generally hyperopic;^58^ therefore, a method to compensate for their refractive error and, at the same time, ensure proper focus control of the STOC-T retinal image on a specific retinal layer or vascular bed of interest is very important. In our system, we implemented a sample interface that can compensate for the mouse eye refractive error by adjusting the distance of the mouse eye to a stationary 0 D meniscus lens in lubricant eye gel, as in the work of Zhang et al.^53^ As soon as the meniscus lens is no longer preceded and followed by the same medium, i.e., as soon as it is in contact with the gel on the mouse side, it becomes a positive lens. Therefore, changing the axial position of the mouse’s eye makes it possible to change the thickness of the gel and bring the retinal layer of interest into focus on the STOC-T camera.

**Fig 2:**
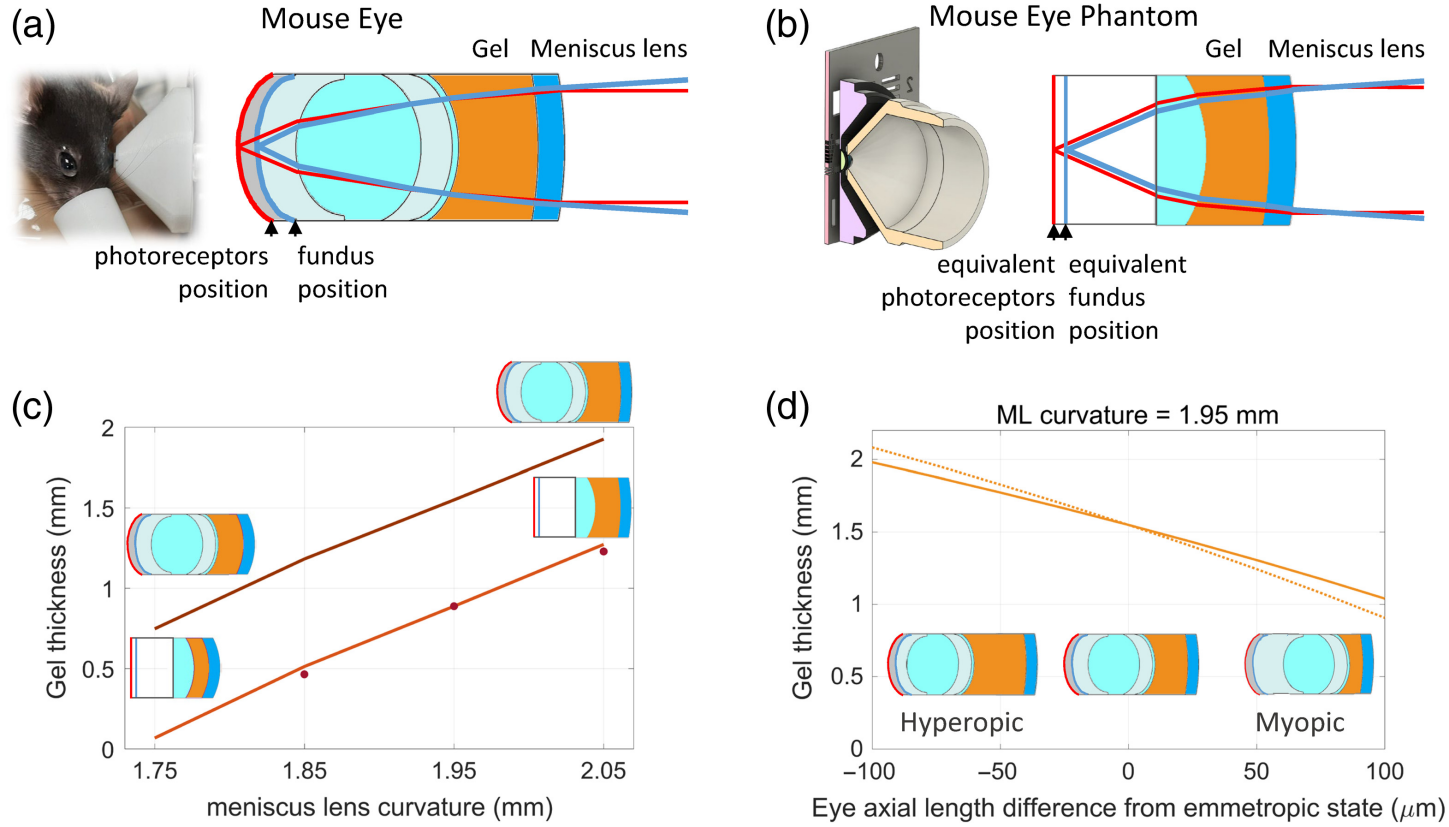
Examples of the appropriate gel thickness to produce a focused image of the photoreceptor layer on the STOC-T camera were obtained with ray tracing optical simulations for different cases. (a) Model of a mouse eye.^59^ (b) Model of a mouse eye phantom, described in detail in Appendix A, where the distance of the plane representing the phantom fundus from the mouse eye phantom focal plane has been calculated to have the same ray vergence as the rays originating from the fundus in the mouse eye model in panel (a), considering the respective wavelengths of interest. (c) Gel thickness as a function of the meniscus lens (ML) curvature for the mouse eye and mouse eye phantom models, where the dots represent the experimental validation data for the latter. (d) Gel thickness as a function of mouse eye refractive error. The dotted line represents the curve for the appropriate gel thickness if we could set it by directly ensuring the correct focal position on the photoreceptor layer from the live STOC-T image. The solid line represents the gel thickness we would set by assessing proper focusing of the mouse fundus on the calibrated fundus imaging system.

The appropriate gel thickness depends on the meniscus lens curvature, with smaller radii of curvature (i.e., more curved meniscus lenses) requiring less gel. Similarly, for a given meniscus lens curvature, myopic eyes (i.e., eyes with a longer axial length than normal) require less gel than emmetropic eyes (i.e., eyes with no refractive error), and hyperopic eyes (i.e., eyes with shorter axial length than normal) require more gel than emmetropic eyes. Figure 2 graphically shows the appropriate gel thickness to have a focused image of the photoreceptor layer in ray tracing optical models of our system and of a mouse eye^59^ [Fig. 2(a)] and the mouse eye phantom, described in detail in Appendix A [Fig. 2(b)], as a function of meniscus lens curvature [Fig. 2(c)], and as a function of the mouse refractive error [Fig. 2(d)]. For reference, the emmetropic eye axial length was 3.4 and 2.31 mm in the mouse eye and the mouse eye phantom ray tracing models, respectively.

### 2.5 STOC-T Image Processing and Digital Aberration Correction

STOC-T image reconstruction and processing followed the pipeline described in detail in our previous work.^31^^–^^33^ However, the STOC-T processing pipeline differed slightly depending on whether or not DAC was incorporated.^50^

In summary, STOC-T volumes were reconstructed by applying the following algorithmic sequence: MM speckle noise reduction by *en face* spatial frequency filtering, selection of the region of interest in depth (*Z*), numerical dispersion compensation, fast Fourier transformation along the wavenumber axis (i.e., the time axis), and normalization by the noise floor.

If no DAC was required, the next step was volume flattening within each volume in the dataset, applied by detecting Bruch’s Membrane (BrM) depth and by co-registering all A-scans to it. Volume co-registration was the following step, whereby all volumes in the dataset were co-registered to the first volume, initially in *Z* to BrM depth and then in the *X* and *Y* planes.

If DAC was required, the processing applied to the reconstructed volumes was as follows. The complex data of each reconstructed volume were 2D-Fourier transformed at each depth *Z*, to obtain the spatial spectrum. This was multiplied by a complex exponential function of an adjustable parameter $\alpha_{n}^{l}$ and a Zernike polynomial $Z_{n}^{l}(k_{x},k_{y})$. The resulting product was then 2D-inverse-Fourier transformed to obtain a phase-corrected reconstructed *en face* image. Several values of the parameter $\alpha_{n}^{l}$ are tried until the kurtosis ξ(α), an image sharpness metric, of the *en face* image intensity is maximized. In the simplest form, only the defocus Zernike polynomial is considered (with indices l=2 and n=0). Alternatively, at the expense of a higher computational demand, up to 22 Zernike polynomials for each *en face* image have been considered.

Once each volume in the dataset underwent DAC, a 3D (X,Y, and Z) co-registration procedure was applied so that all volumes in the dataset were co-registered to the first one. This was followed by volume flattening.

### 2.6 Phase Stability of the System

The mouse eye phantom was also used to determine the phase stability of the STOC-T system by evaluating the best inter-layer displacement sensitivity, i.e., the sensitivity to displacement over time between two depth layers in the same volume, for phantom layers presenting a similar signal-to-noise ratio (SNR) to, for example, the mouse NFL layer and the BrM. To do so, two layers of adhesive tape were laid on top of the USAF test target, to create closely spaced layers of realistic SNR.

Equation (1) computes the STOC-T theoretical inter-layer displacement sensitivity from the theoretical inter-layer phase difference sensitivity $\sigma_{\Delta \Delta \varphi }$, where the ΔΔ symbol indicates a double phase difference operation, i.e., between layers and over time, as

$$d_{\Delta \Delta \varphi }=\lambda_{0}\frac{\sigma_{\Delta \Delta \varphi }}{4\pi }.$$ (1)

The theoretical inter-layer phase difference sensitivity $\sigma_{\Delta \Delta \varphi }$^60^ is inversely related to half the composite SNR, ${\mathrm{SNR}}_{c}(z_{1},z_{2},t)$, arising from the different SNRs of the two retinal layers of interest, $\mathrm{SNR}(z_{1},t)$ and $\mathrm{SNR}(z_{2},t)$, where ${\mathrm{SNR}}_{c}(z_{1},z_{2},t)\;=\frac{2\mathrm{SNR}(z_{1},t)\mathrm{SNR}(z_{2},t)}{\mathrm{SNR}(z_{1},t)+\mathrm{SNR}(z_{2},t)}$. It follows from Eq. (2) that

$$\sigma_{\Delta \Delta \varphi }=\sqrt{\frac{2}{{\mathrm{SNR}}_{c}(z_{1},z_{2},t)}}=\sqrt{\frac{\mathrm{SNR}(z_{1},t)+\mathrm{SNR}(z_{2},t)}{\mathrm{SNR}(z_{1},t)\mathrm{SNR}(z_{2},t)}}.$$ (2)

The SNR of the top of the air-tape interface of the top layer was $\mathrm{SNR}(z_{1},t)=∼20\;\mathrm{dB}$, whereas the SNR of the interface between the two tape layers was $\mathrm{SNR}(z_{2},t)=∼23\;\mathrm{dB}$. Therefore, the composite STOC-T SNR from the two layers is 21.4 dB for the central (x,y) position in the FOV. The theoretical inter-layer displacement sensitivity is 27.6 nm. The corresponding experimental inter-layer displacement sensitivity was 26.2 nm, where reduced adhesiveness between tape layers over time, t, might have contributed to an increase in $\mathrm{SNR}(z_{2},t)$, leading to a result slightly better than expected. Moreover, at the cost of reducing the lateral resolution, magnitude averaging among areas of N (independent) pixels could improve the SNR by a factor of $\sqrt{N}$.^61^ For a local average in a central region of 15 by 15 lateral pixels and three depth pixels, the measured STOC-T inter-layer displacement sensitivity further improved to 1.2 nm. This size region, corresponding roughly to 16×14  μm, is the one we chose for phase-sensitive hemodynamics measurements.

We also evaluated the displacement sensitivity of the system during an *in vivo* measurement of a mouse retina as this is a more relevant parameter. However, several factors render this measurement complicated. First, the mouse eye moves slightly during the acquisition, and therefore, it is important that the volume co-registration algorithm works well. Even in such a case, we can expect that the mouse motion might have introduced some additional noise to the phase-sensitive measurement. Second, in an *in vivo* measurement, the retinal layer (NFL–BrM) displacement induced by the blood pulse wave, i.e., one of the parameters we would like to quantify for hemodynamic analysis, is present on top of the noise floor we want to evaluate for a displacement sensitivity assessment. Therefore, to minimize the pulsation-induced retinal displacement influence, we subtracted the intra-layer displacement over time between neighboring regions and obtained an equivalent inter-layer displacement sensitivity of 5.8 nm.

### 2.7 Hemodynamics Analysis

We performed two types of hemodynamics analysis. The first one derives from the computation of the spatially resolved STOC-T signal amplitude variation over time, which is useful for highlighting the position of blood vessels forming the retinal and choroidal vasculature. The comparative analysis of the temporal pulse traces from different vessels and from different locations along some vessels enables the characterization of phase differences (delays) between arterial and venous pulsation and pulse wave velocity (in plane), respectively. In addition, STOC-T axial sectioning enables this comparative analysis for different vascular beds in the chorioretinal complex.

The second type of analysis derives from the computation of the spatially resolved STOC-T phase among different retinal layers (*en face*) over time. By choosing the NFL as the layer of interest and the BrM as the reference layer, one can measure the retinal tissue displacement in the order of tens to hundreds of nanometers over time due to pulse wave propagation. More specifically, we calculate the phase differences over time to the first time point for the NFL and BrM for a range of 3-depth pixels. Then, we calculate, pairwise, the phase difference between NFL and BrM and average it over the depth dimension. Then, we apply Gaussian filtering with a kernel parameter σ equal to 5 pixels. We mask out regions of low STOC-T amplitude. Then, we first perform lateral phase unwrapping,^62^^,^^63^ followed by phase unwrapping in time. We cut out phase difference values exceeding ±1.5π, corresponding to wrapping artifacts around and above vessels. We then translate phase differences to tissue displacement, using Eq. (1).^60^

## 3 Results

**Fig. 3:**
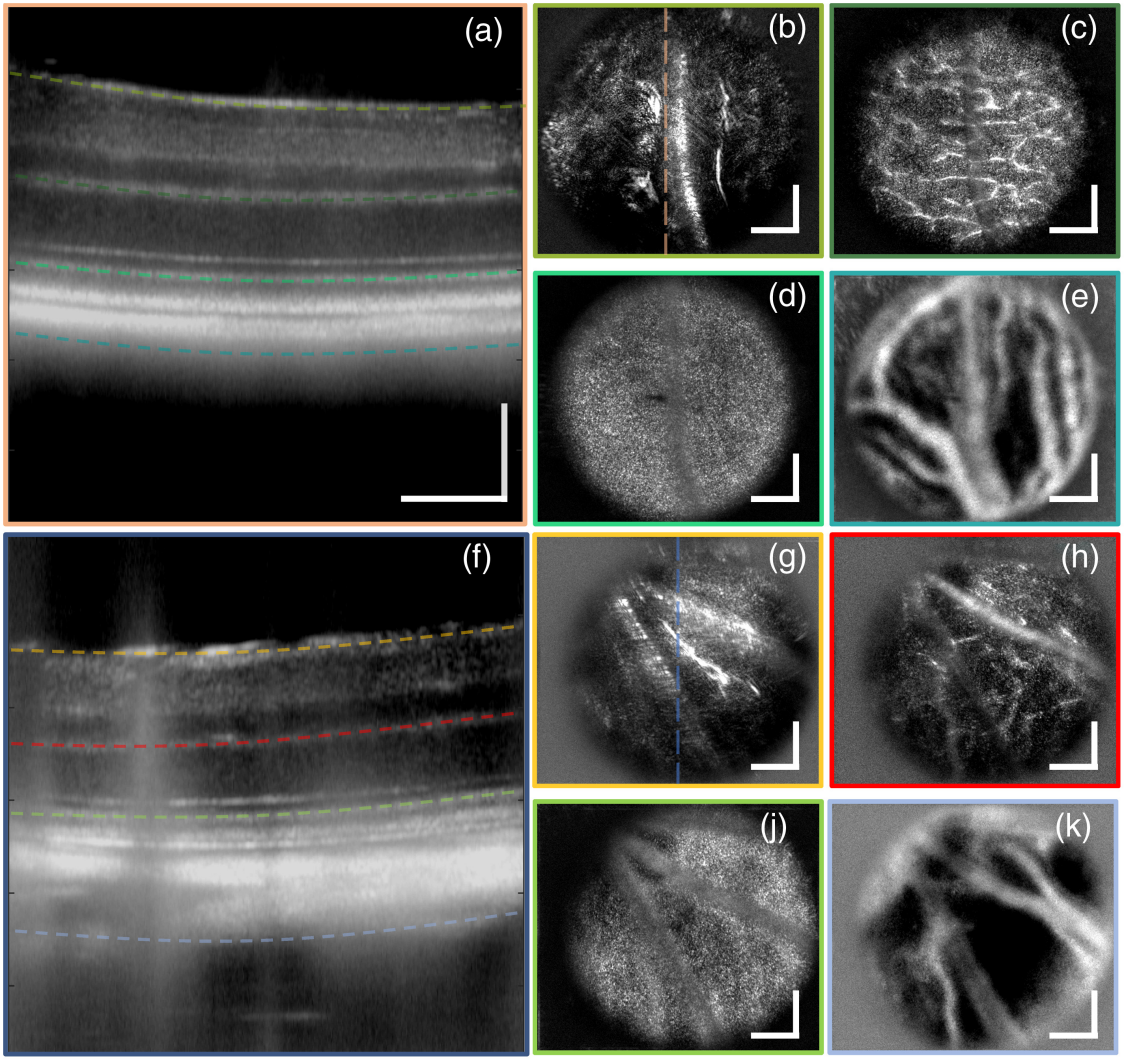
Cross-sectional and *en face* images from STOC-T volumetric scans of the mouse retina for a wild type mouse (a)–(e), and albino mouse (f)–(k). The location of *en face* images of (b), (g) the NFL, (c), (h) the IPL, (d), (j) the IS/OS junction, and (e), (k) the choroid is indicated in either panel (a) or panel (f) by the dashed color line with matching color to the *en face* image contour. The scale bars represent 100  μm.

Figure 3 shows examples of the type of volumetric structural images of the mouse retina and choroid that can be obtained with our high-speed STOC-T system. Figures 3(a)–3(e) are images from a wild-type mouse retina, whereas Figs. 3(f)–3(k) are images from an albino mouse retina. All images are the results of magnitude averaging from 60 co-registered DAC-processed volumes acquired in 0.532 s. DAC took place by correcting the first 22 Zernike terms. Figures 3(a) and 3(f) present an average of 10 central B-scans, highlighting the layered structure of the mouse retina. In both, dashed color lines at increasing depth in the retina indicate, in order, the location of the NFL, the inner plexiform layer (IPL), and the photoreceptor inner and outer segment (IS/OS) junction and the choroid, which are shown in *en face* images boxed with the corresponding color in Figs. 3(a) and 3(f). The NFL images [Figs. 3(b) and 3(g)] and the IPL images [Figs. 3(c) and 3(h)] are the result of a 10-depth pixel average; the IS/OS images [Figs. 3(d) and 3(j)] are the result of a 20-depth pixel average; and the choroidal images [Figs. 3(e) and 3(k)] are the result of a 65-depth pixel average.

Importantly, larger blood vessels are visible in both the superficial layers, e.g., the NFL, and in the choroid, whereas a more intricate network of smaller vessels and capillaries is visible in the IPL. The IS/OS junction image reveals a speckle pattern reminiscent of the underlying photoreceptor mosaic, although not fully resolved, as resolving individual rods in mice proved to be complicated if not elusive to AO techniques too.^46^^,^^47^^,^^64^

**Fig. 4:**
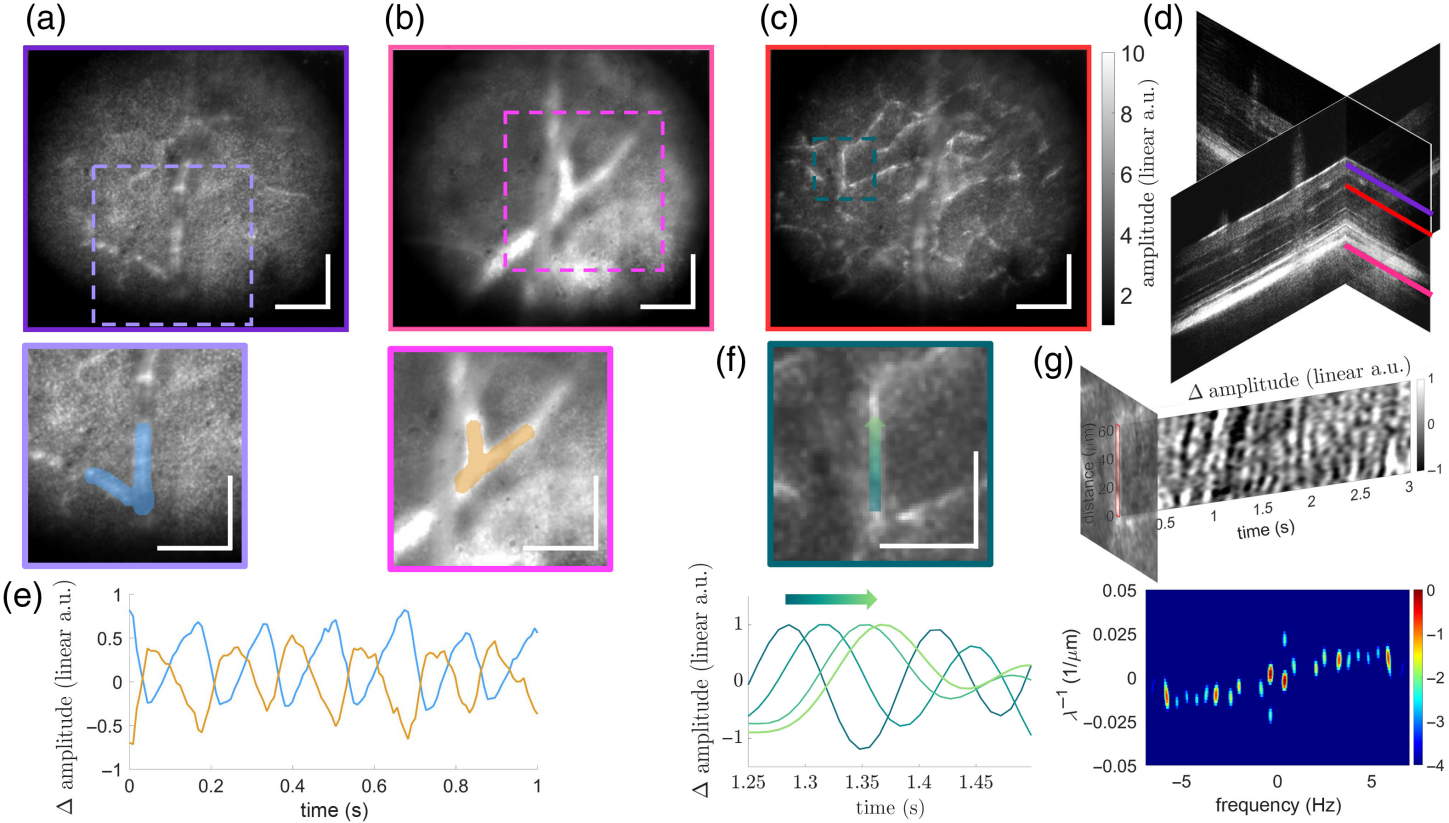
Mouse chorioretinal hemodynamics analysis in the NFL, OPL, and the choroid, based on STOC-T amplitude signal variation over time in an albino mouse. STOC-T *en face* images of (a) the NFL and (b) the choroid with zoomed-up ROIs shown just below, where the shaded areas highlight blood vessels. STOC-T *en face* image of (c) the OPL, with indicated ROI. Scale bars represent 100  μm. (d) The relative depth of the analyzed layers (NFL, OPL, and choroid) within orthogonally sliced cross-sections of the STOC-T volume (shown in intensity dB). (e) Deviation from the mean pixel amplitude over time, averaged within the shaded areas in (a) cyan and (b) orange, in the respective ROIs. (f) OPL ROI with a green gradient arrow overlaid on a capillary, indicating the direction of blood flow. Below is a graph of the STOC-T signal amplitude variation over time, normalized to each maximum, for different locations along the highlighted vessel (as indicated by the color gradient), showing the propagation of the pulse wave. (g) Time-space representation (top) of the variation from the mean amplitude in time along the vessel region highlighted in (f) with corresponding 2D Fourier transform amplitude (bottom), similar to the dispersion relation graph, presenting different frequency components propagating at a group velocity of ∼0.35  mm/s. The plot uses a logarithmic scale normalized to the maximum value.

In the following steps, we extracted hemodynamics information, captured by the high-volume rate of the system, by visualizing how the amplitude of STOC-T changes at different depths over time. The results are depicted in Fig. 4. In this hemodynamics study, we specifically focused on a single mouse model to maintain consistency and provide a clear demonstration of retinal hemodynamics using the STOC-T system. Investigating subtle differences between mouse types requires detailed quantitative analysis within one model to establish a baseline, which can then serve as a reference for future comparative research.

Figures 4(a)–4(c) show STOC-T *en face* images of an albino mouse retinal layer just below the NFL, a layer in the choroid, and the outer plexiform layer (OPL), respectively, generated by magnitude averaging in the depth-ranges indicated in Fig. 4(d) and then also averaging in time (340 volumes, 3.0147 s). Figure 4(e) presents the deviation from the mean pixel amplitude in time, averaged in small regions of blood vessels, just below the NFL and in the choroid, in cyan and orange color, respectively. These regions, including blood vessels branching, are highlighted by the shaded areas in the zoomed-up regions of interest (ROIs) in Figs. 4(a) and 4(b), respectively. A moving average filter with a five-sample window (44.3 ms) has been applied to smooth the curves. An interesting observation is that the mouse retinal superficial vessel and choroidal vessel pulsate both with a frequency of roughly 6 Hz but are nearly in phase opposition, corresponding to a delay of ∼83  ms. Figure 4(f)—top—presents the ROI in the OPL with a green gradient arrow overlaid on a capillary, indicating the direction of blood flow. The flow could be observed with the time resolution of our system, and the group velocity of the pulse wave could be assessed. Figure 4(f)—bottom—presents a graph of the STOC-T signal amplitude variation over time for four different locations along the highlighted vessel (as indicated by the color gradient), with each curve first low-pass-filtered with a cut-off frequency of 6.25 Hz, averaged in a small region of the vessel, smoothed by the same moving average filter as in Fig. 4(e), and then normalized by each maximum to emphasize the wave propagation characteristics. It is evident that the pulse signal in such a small vessel is not as regular and it presents a broader bandwidth than the signal for the larger superficial and choroidal vessels. However, it can be noted that the peaks and throughs of the pulse temporal waveform move along the capillary at a quantifiable speed. This is even clearer from Fig. 4(g)—top—showing STOC-T signal amplitude variations averaged across the vessel thickness and shown as a map plotting time versus distance along the vessel direction. A slanted pattern is noticeable in this map, confirming a repeated observation of capillary blood flow. Figure 4(g)—bottom—allows us to estimate the group velocity of this pulse wave. In fact, it shows the corresponding 2D Fourier transform amplitude, presenting different frequency components grouped along a constant spatial frequency—temporal frequency ratio, corresponding to a specific propagation velocity, which can be estimated as ∼0.35  mm/s.

Video 1 shows a time sequence of STOC-T *en face* amplitude images, averaged through the depth-ranges indicated in the sliced volume cross-sections in Fig. 4(d). On the right, the same three layers are shown as a time sequence of the averaged *en face* images following Gaussian filtering, amplitude squaring, and temporal mean removal. These extra processing steps aid the visualization of blood vessel pulsation and pulse wave propagation as temporal black-to-white fluctuations over a rather constant gray background. Interestingly, we can observe how the pulse wave group velocity in larger superficial and choroidal vessels is too high for the system to detect, and the whole vessel within the FOV pulsates almost at the same time. This is in contrast to the smaller vessels and capillaries of the OPL, where the pulse wave propagation can be sampled with the high volumetric temporal resolution of our system.

Video 2 shows the time sequence of the same dataset as in Video 1, for the NFL and choroidal layers and also for a B-scan cutting through a superficial vessel just below the NFL. This is useful to highlight how blood flow affects signal in depth in STOC-T. In fact, we can observe that the signal fluctuates not only at the vessel location depth but also above and below the vessel. In the graph, we plot the STOC-T amplitude over time for the average amplitude within the cyan and brown regions, corresponding to the location of the branching of the superficial vessel itself, and the same lateral location deep in the choroid, where no major vessel is visible, respectively. The signals were additionally filtered using a moving average filter with a five-sample window length. It is evident how the cyan and brown curves are opposite in phase as they mirror each other, i.e., once the signal in the superficial vessel peaks, the signal from the vessel projection in the choroid is at its minimum, and vice versa. By observing the B-scan, we can see that the signal appears to periodically concentrate around the superficial vessel and then deplete from the vessel location while smearing to neighboring depths, both below and importantly above, i.e., at shorter optical pathlengths. In Sec. 4, we elaborate on the likely cause of this phenomenon. Nevertheless, Video 2 confirms that the choroidal vessel pulsation is delayed from the superficial vessel pulsation, even when the lateral positions of the two vessels do not match. This allows us to rule out any artifactual nature of this delay, despite the fact that the phase offset between the two is incidentally in opposition, as reported also in Fig. 4(e). Therefore, this hints at differences in arterial versus venous vessel types.

**Fig. 5:**
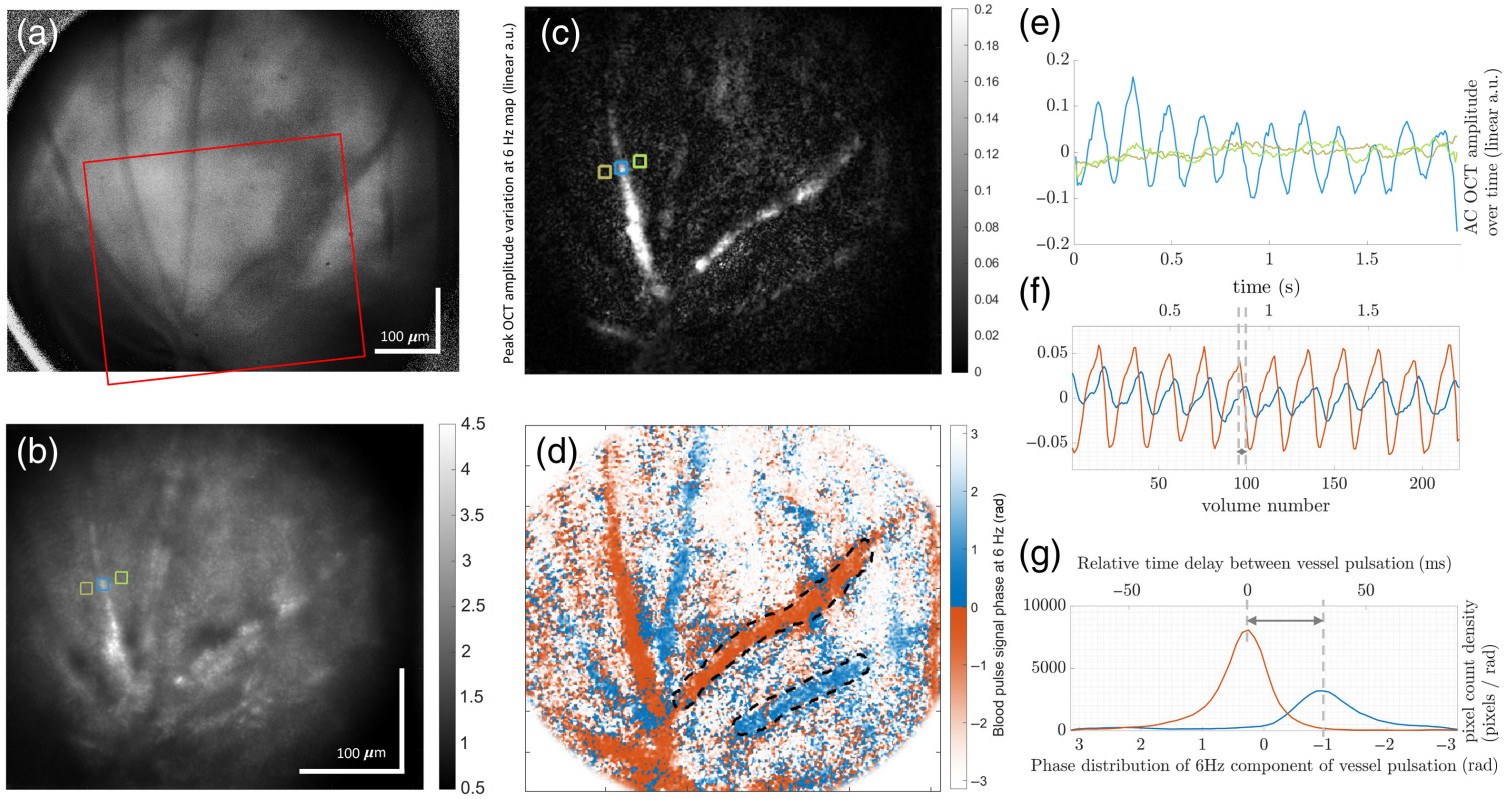
Characterization of venous and arterial pulsation in an albino mouse retina through STOC-T amplitude analysis. (a) An image acquired with the fundus camera showing the STOC-T image FOV inside the red box. (b) STOC-T depth-averaged *en face* image of the NFL via a maximum amplitude projection through time. (c) Spatial distribution of the amplitude of the STOC-T temporal variation signal spectrum at a frequency of 6 Hz (the mouse heartbeat rate). (d) Spatial distribution of the corresponding phase of the 6 Hz signal component, revealing temporal differences attributable to the delay between arterial and venous pulsation. (e) Mean-subtracted time series of STOC-T amplitude averaged within three small boxes marked in panel (c). (f) Mean-subtracted time series of STOC-T amplitude averaged inside the regions bounded by the two dashed black lines in panel (d). The average peak delay between the pulse traces equals 29 ms. (g) Normalized histogram of pulse trace phase values. The temporal separation between the arterial (orange) and venous (light blue) distributions confirms the results shown in panel (f).

We continued the analysis of mouse retinal hemodynamics by distinguishing between venous and arterial pulsation in the mouse retina through STOC-T amplitude analysis. Figure 5(a) shows an image of an albino mouse retina acquired with the fundus camera providing an overview of the retinal region studied with the STOC-T system (outlined by the red box). In this case, out of the 340 volumes, 221 happened between mouse breathing events, so we focused on this more stabilized subset of volumes. The processing included DAC for defocus correction. Figure 5(b) presents a maximum amplitude projection of a 20-layer averaged *en face* image of the NFL, through time (i.e., through 221 STOC-T volumes). The vessel visibility is hindered by the surrounding scattering regions as it is common also in flying spot OCT. However, the high temporal resolution allows us to sample the blood flow pulsation-induced STOC-T amplitude signal variation and localize it to the vasculature outline. Figure 5(c) shows the *en face* image of the superficial vasculature by plotting the band-pass (0.5 Hz bandwidth) filtered amplitude of the signal spectrum at the heartbeat rate of 6 Hz. Figure 5(d) presents the phase of the 6 Hz signal component, revealing temporal differences attributable to the delay between arterial and venous pulsation. All phases are shifted by constant component (1.3π  rad) to keep the distribution around 0 value for presentation purposes. Figure 5(e) shows the mean-subtracted time series of the STOC-T amplitude averaged within three specific 15 by 15-pixel regions marked in Fig. 5(c), filtered with a moving average of nine samples (79.8 ms window). The curves highlight the predominant contribution of blood vessels (cyan curve) to the observed STOC-T amplitude oscillations (compared with the green curves from just outside the vessel). We evaluated the delay between the pulse traces of two vessels in the areas marked by a dashed black line in Fig. 5(d). We reported the average peak delay to be 29 ms in Fig. 5(f), showing mean-subtracted time series of the STOC-T amplitude averaged inside the regions bounded by the two dashed black lines in Fig. 5(d), filtered with a moving average of five samples (44.3 ms window). Figure 5(g) presents a normalized histogram (pixel counts per radian) of the phase values of pixels within the regions bounded by the two dashed black lines in Fig. 5(d) (with bin width 0.1 radians, and the pixel counts smoothed with a moving average filter of a 5-bin window length). The temporal separation between these two distributions confirms the results shown in Fig. 5(f). The exquisite temporal resolution of our STOC-T system enabled the classification of arterial and venous pulsation, delayed by only 29 ms, with the arteries (in orange) pulsating before the veins (in light blue).

**Fig. 6.**
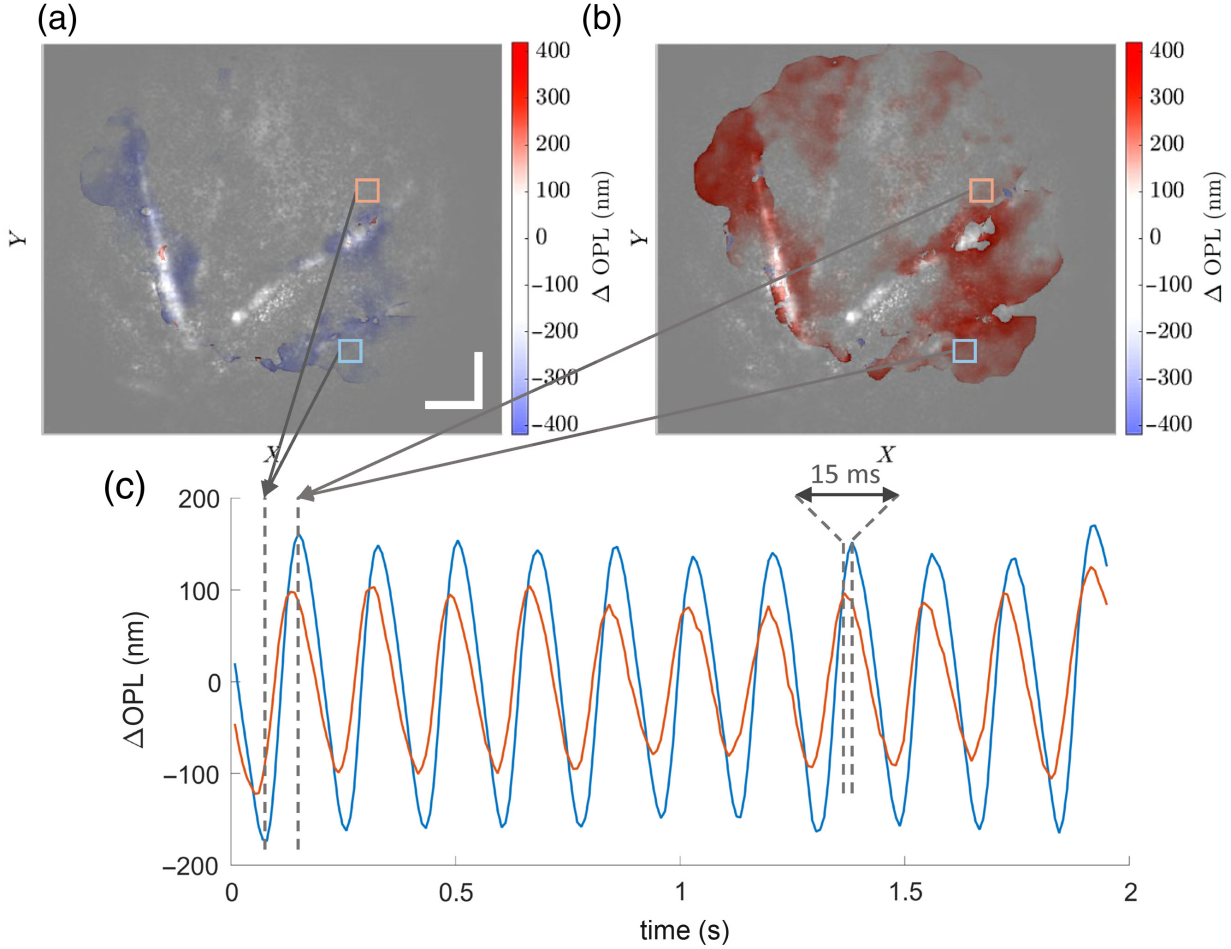
Retinal tissue displacement caused by vascular dynamics from the analysis of STOC-T phase difference between NFL and BrM over time. (a), (b) Tissue displacement maps separated by 80 ms, i.e., roughly by half heartbeat cycle (period), overlaid to the filtered amplitude of the STOC-T signal spectrum at 6 Hz, as in Fig. 4(c). Scale bars represent 50  μm. (c) Time plots of tissue displacement at two chosen locations, near an artery (pink box) and a vein (light blue box), indicated in panels (a) and (b).

Last, we extracted additional hemodynamics and biomechanics information, captured by the high-volume rate of the system, by visualizing how the phase difference among different layers (depths) in a STOC-T volume changes over time. The results are depicted in Fig. 6.

Figures 6(a) and 6(b) present tissue displacement maps, at 44 and 124 ms from the start of the STOC-T recording, respectively, separated roughly by half heartbeat cycle (period), overlaid to the filtered amplitude of the STOC-T signal spectrum at 6 Hz, as in Fig. 4(c). We can observe that the tissue responds to a pulsating vessel by expanding and contracting in sync with the pulse wave and how this displacement is mostly, but not entirely, concentrated above and around the vessels. In Fig. 6(c), we show the time plots of tissue displacement at two chosen locations, near an artery (pink box) and a vein (light blue box). To generate those plots, first, we average the tissue displacement over the chosen square region of 15 by 15 pixels, and then, we filter out signal frequency components below 2.5 Hz and apply a moving average filter with a five-sample window length. The average displacement modulation amplitude is roughly 100 and 150 nm near the artery and the vein, respectively. Also, a characteristic delay is present, albeit smaller than in corresponding Fig. 4(f). We calculated the delay between the two plots as the mean difference between corresponding signal peaks, and it was 15 ms.

Video 3 presents a time sequence of both STOC-T *en face* amplitude images of the NFL of the mouse retina presented in Figs. 5 and 6 and an overlay of the corresponding mean-subtracted time sequence with the tissue displacement map. This visualization compiles complementary information about the spatially resolved hemodynamics and biomechanics analysis we can perform on the mouse retina.

## 4 Discussion

The origin of the STOC-T amplitude variation with blood pulsation can be explained by the Doppler broadening^65^^,^^66^ caused by turbulent erythrocyte flow within the vessel^18^ (especially in the STOC-T axial direction) during the comparatively slow wavelength sweep (8.5 ms). During systole (when the cardiac muscles contract and blood pressure is maximum), the speed of circulating red blood cells is higher, and the corresponding Doppler broadening is larger than during diastole (when the cardiac muscles relax and the blood pressure is the lowest). As a result, the STOC-T signal at the depth where the blood vessel is located is at a minimum during systole as the signal is artifactually spread across a wider depth range, whereas it is at a maximum during diastole as the Doppler spread is reduced. However, the opposite trend happens at large distances below (and above) the blood vessel. In fact, systole-induced stronger Doppler broadening leaks noise onto the underlying signal, effectively leading to a maximum of the local STOC-T amplitude, whereas the added noise recedes during diastole, as it can be seen in Video 2. So, despite being artifactual in nature, the Doppler signal broadening variation gives us a surrogate signal to track blood pulse traces and flow velocities in capillaries. In addition, it allowed us to distinguish the arteries from the veins and identify the arteries themselves as they give a stronger signal than the vein during systole because of the higher speed of circulating red blood cells, which is in agreement with the results reported in the literature.^18^

The pulse wave velocity (in an *en face* plane) was too high to be detected in the larger superficial and choroidal vessels, but we could still detect it and measure it in the capillaries of the OPL. We measured a mean blood pulse group velocity of 0.35 mm/s, which is consistent with the literature, reporting mean blood flow velocities in the capillaries of the human retina to be below 3 mm/s^67^^,^^68^ and around 0.5 mm/s in the capillaries of the mouse retina.^69^ In our system, the upper bound for measurable pulse wave velocities in a straight vessel is ∼50  mm/s. At the detected mouse heartbeat frequency of 6 Hz, this velocity should come from a pulse wave with a wavelength of ∼8  mm, which is much larger than our FOV. Ultimately, the pulse wavelength might be a stronger limitation to measuring velocities in larger vessels than the temporal resolution of our system. Even for velocities in the order of 23 mm/s, i.e., the highest mean velocity in mouse retinal vessels reported in Ref. 69, the wavelength would be roughly 10 times the STOC-T FOV, making it hard to identify a crest (of the Doppler artifact) within a vessel, and there would only be two time points before that crest would disappear from the FOV. Nonetheless, we believe that our system is a powerful tool for monitoring pulse wave velocities in arterial and venous microvasculature in the retina and deep in the choroid.

Moreover, our system can leverage its phase-sensitive ability to detect nanometer-range tissue displacement due to the traveling pulse wave. We noticed how tissue displacement above and near major vessels had modulation amplitudes between 100 and 150 nm, potentially carrying information about the biomechanical properties of the retinal layers involved. We also identified a delay in tissue displacement above a vein from the displacement above a neighboring artery. However, this delay (15 ms) was smaller than the delay measured from the analysis of the Doppler-induced STOC-T amplitude modulation (29 ms). This discrepancy could be in part due to slightly different response times of the tissues involved and their viscoelastic properties, but, more likely, to a phase-sensitive STOC-T measurement integrating contributions from several possible sources of tissue displacement beyond that of just the vessels of interest themselves. In fact, by calculating the displacement between Bruch’s membrane and the NFL, pulsation-induced motion at any depth between these two could contribute an out-of-phase component to the overall phasor sum, reducing the apparent delay between the two superficial pulsation-induced displacements.

Last, in future studies, it will be beneficial to exchange the visible light illumination for the fundus imaging system with an IR LED to avoid retinal stimulation with visible light for studies where visual stimulation needs to be included, including long periods of dark adaptation, compatible only with an IR fundus imaging system.

## 5 Conclusions

In summary, we demonstrated an experimental setup for mice retinal imaging based on the STOC-T technique, with a fundus camera and white light illumination aiding the alignment of the mouse. We presented *en face* images of the retinal and choroidal microstructure from various layers. The described experimental design facilitates the high-speed acquisition of high-quality, *in vivo* volumetric mouse retina imaging at a rate of 113 Hz and provides insight into the hemodynamics of the mouse retina and choroid that could be beneficial in the study of neurovascular coupling and vasculature and flow speed anomalies in neurological and neuro-ophthalmological conditions.

## 6 Appendix A: Mouse Eye Phantom

A mouse eye optical phantom has been developed to calibrate the mouse retinal volumetric imaging system, to test its performance, and to measure several system specifications. The mouse eye phantom consists of a visible-NIR antireflection-coated plano-convex lens (#65-300, Edmund Optics), with a diameter of 2.5 mm and an effective focal length of 2 mm, glued on a custom-made 3D printed mount [see Fig. 2(b)]. The lens clear aperture and effective focal length resemble those of a mouse eye.^70^ A 2" × 2" negative, USAF 1951 high-resolution target (#55-622, Edmund Optics) mounted on an X,Y,Z, and tip-tilt stage served as a phantom imaging plane, which could be set at the focal distance from the mouse eye phantom lens, simulating an emmetropic mouse retinal photoreceptor layer, or at different axial distances, simulating ametropia. Due to the lack of layered structure of the mouse eye phantom “retina,” we were using the USAF test target at two given positions, representing the photoreceptor and fundus layers, respectively, for any given mouse eye refractive error simulation. Through the help of ray tracing software (Zemax OpticStudio, Ansys, Canonsburg, United States), we determined the forward shift of the USAF test target from the position of the simulated photoreceptor layer needed to simulate the corresponding mouse eye fundus position. The whole mouse eye phantom was placed on X,Y, and Z stages, allowing to solidly move the eye phantom toward or away from the meniscus lens, with the mouse eye phantom lens able to get almost in contact with the meniscus lens within the mildly viscous gel.

The mouse eye phantom was used to determine the lateral resolution and FOV of the imaging systems. The measured lateral resolution was 1.74 and 1.23 μm (corresponding to the last resolvable element of the USAF test target being the second and fifth elements from the eighth group), for the STOC-T and fundus imaging systems, respectively.

## 7 Appendix B: Imaging Systems Focal Offset Calibration Procedure and STOC-T Focus Fine Tuning

Proper gel thickness selection and mouse alignment are crucial for obtaining good-quality STOC-T images. The best way to ensure proper mouse eye alignment and focusing on the retinal layer of interest before starting an STOC-T volumetric sequence acquisition would be by using a live STOC-T image preview combined with a proper precision optomechanical apparatus to align the mouse during live preview.

Using the selected high-speed camera (Nova S-16, Photron), we encountered difficulties in implementing live preview due to the extended duration (∼400  ms) needed to switch the camera from the recording to the data transfer mode. This transition time resulted in a live preview refresh rate of ∼1.5  Hz, which motivated us to incorporate a mouse fundus imaging system to provide a real-time preview.

Moreover, as the NIR illumination is roughly collimated and STOC-T lacks the confocal effect by design, the variation in STOC-T signal-to-noise ratio (SNR) due to proper focusing is more subtle than that in standard flying-spot OCT; therefore, a visual evaluation of the B-scan intensity is not sufficient to accurately determine where the focus lies.

The mouse fundus imaging system facilitates live preview and fine-tuning of the mouse position to adjust the focal plane position in the corresponding STOC-T volume as the evaluation of proper focusing of superficial retinal vessels, the optic nerve head, and possibly nerve fibers is more immediate in the fundus image than in the small FOV, low refresh rate STOC-T B-scans. The system works as follows: by adjusting the mouse position and the corresponding gel thickness until the white-light image of the mouse eye fundus appears to be centered around the region of interest and in focus on the fundus camera, we consequently bring the corresponding NIR STOC-T image of the mouse eye photoreceptor layer also in focus on the STOC-T camera (or another layer of interest, as per the following calibration procedure).

**Fig. 7:**
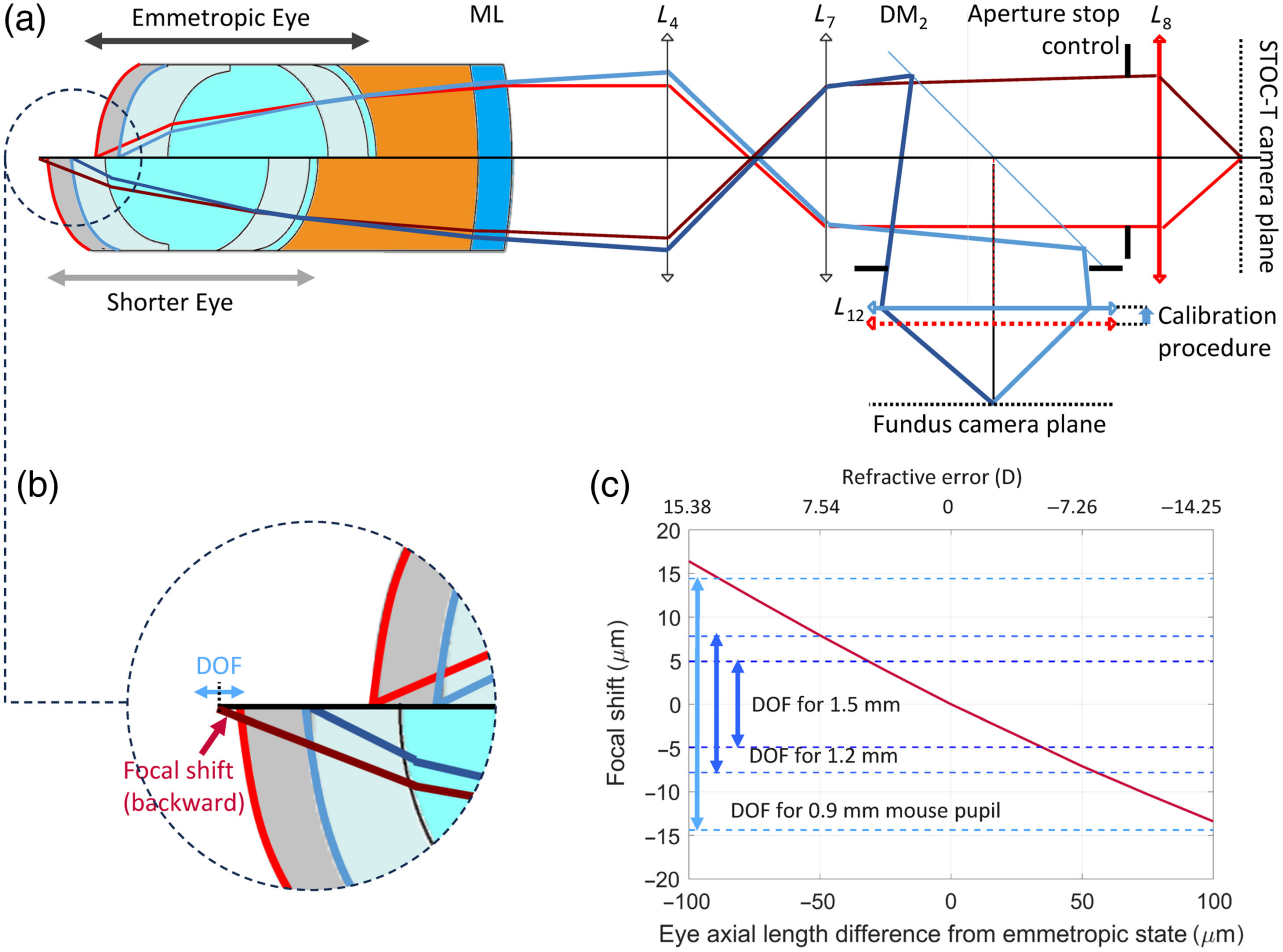
Focal shifts from the photoreceptor layer in STOC-T images resulting from the adjustment of the gel thickness based on the fundus camera image sharpness as a function of mouse refractive error. (a) Optical ray tracing schematic (ray vergence not to scale) showing a vertical split between the case of an emmetropic eye and a hyperopic eye. (b) Close-up on the backward focal shift that occurs in a shorter eye (bottom half) for the STOC-T path when the gel thickness is adjusted by focusing the fundus image on the calibrated fundus camera. The DOF will determine if the photoreceptor layer (or other layer of interest) can still be considered in focus. (c) Graph of the focal shift as a function of axial length variations from the emmetropic state (or equivalent refractive error in D), with the indication of the DOF for several mouse pupil diameters, for the case of a meniscus lens ML curvature of 1.95 mm.

To ensure that this is the case, a focal offset calibration procedure using the mouse eye phantom is performed. The fundus imaging system is calibrated by adjusting the position of lens $L_{12}$ [Fig. 7(a)] so that when the white-light image of the mouse eye phantom “fundus” appears in focus, the corresponding NIR STOC-T image of the mouse eye phantom “photoreceptor layer” is also in focus. As the distance of these equivalent layers in the mouse eye phantom imposes, after the meniscus lens, the vergence difference expected between rays originating from the corresponding retinal layers of an emmetropic mouse eye,^59^ we consider the calibration to be valid also for a generic emmetropic mouse eye.

However, if the mouse eye under inspection presents an (unknown) refractive error, there will be a small disparity between the gel thickness we set by bringing the mouse eye fundus into focus on the fundus camera, and the one that would be required by the STOC-T camera to have the photoreceptor layer in focus [see Fig. 2(d)]. Moreover, this disparity increases with refractive error. Therefore, with the gel thickness set using live feedback from the fundus camera, we might experience a focal shift from the intended layer of interest in the retina in the STOC-T image. Figure 7(a) shows this effect in a vertical split optical ray tracing schematic, where (on the left) the top part represents the case of a mouse emmetropic eye and the bottom part represents the case of a mouse hyperopic eye. For the emmetropic eye, the focal offset system calibration leads, by design, to imaging the intended retinal layer in focus on the STOC-T camera. For a hyperopic eye, in the STOC-T image, there is a focal shift toward a deeper layer than intended [see close-up in Fig. 7(b)].

To assess the severity of this problem, we need to consider both the mouse refractive error and the imaging DOF, and how the latter is affected by the imaging system aperture control from the iris placed after lens $L_{7}$. Basically, we can expect that if the focal shift is within the DOF, the calibration still holds, and we can still reasonably assume we are imaging the photoreceptor layer (or any other calibrated layer of interest) in focus on the STOC-T camera. We present the results of this analysis in Fig. 7(c), where one can see that if we close the iris to an aperture equivalent to a diameter of 1.5 mm in the conjugate mouse pupil plane, the DOF will be ∼10  mm. In this case, the focal shifts from the retinal layer of interest in the STOC-T image would be acceptable only for mouse eye axial length variations of ∼±30  mm from the emmetropic condition. For an equivalent mouse pupil of 0.9 mm, axial length variations up to nearly ±100  mm, i.e., for most mouse eyes (with refractive errors below ±14  D), would produce shifts still within the DOF (∼30  mm). Obviously, reducing the pupil size, while it limits the effect of aberrations in the mydriatic mouse eye, reduces the theoretical (diffraction-limited) lateral resolution. This also means that the resolution limit that can be achieved with DAC is reduced.

In most of the images presented in this paper, we maintained an equivalent pupil size between 1.5 and 1.2 mm. As a matter of fact, after the initial position of the mouse was adjusted based on feedback from the fundus camera, we fine-tuned the position based on the presence of increased speckle contrast due to the pulsation of the layer of interest, in a STOC-T preview screen, showing the raw frame (*X* and *Y*) from the STOC-T camera at a given wavelength.

**Fig. 8:**
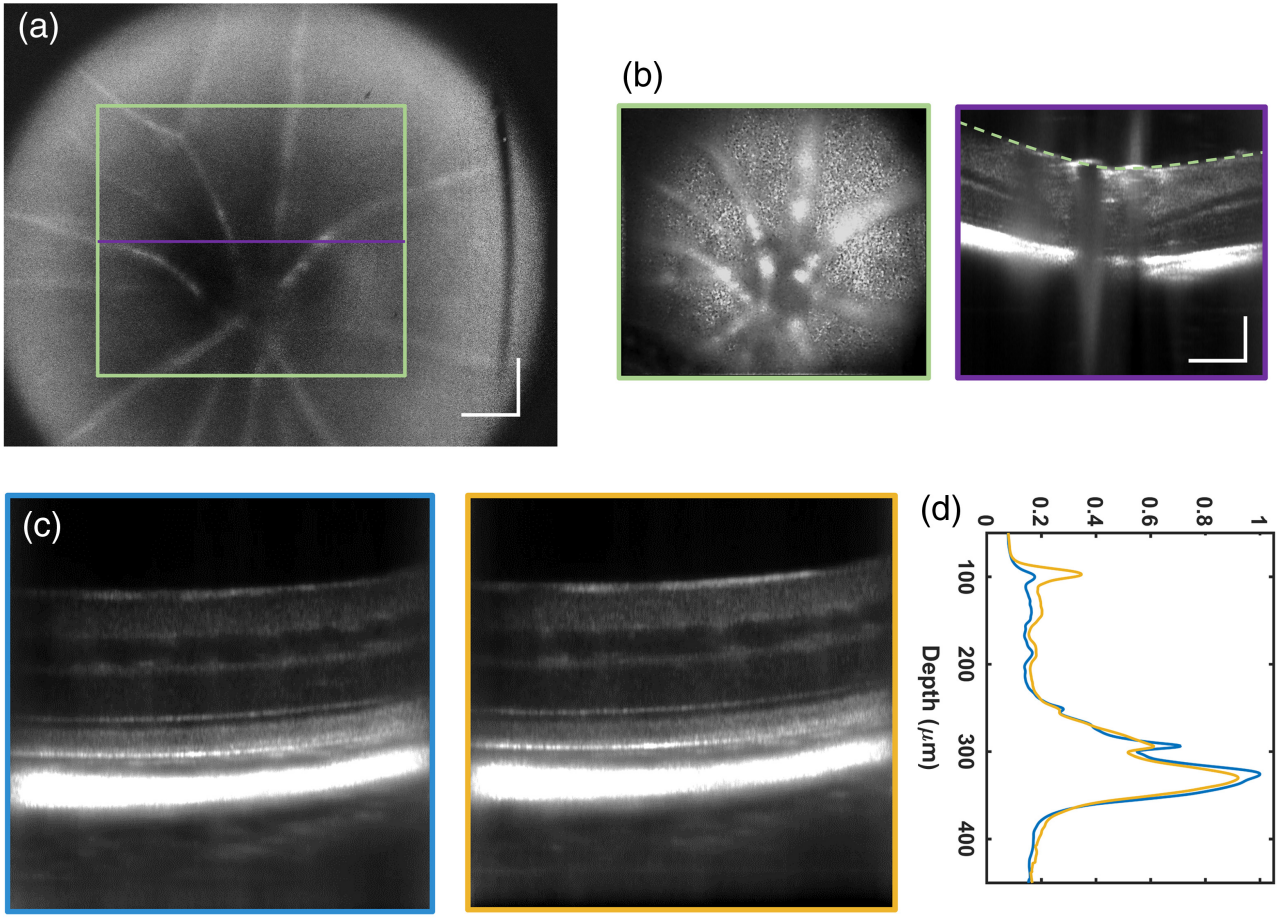
Mouse retinal images show the combined use of a calibrated fundus imaging system for tuning the focus of the STOC-T images. (a) Fundus image centered around the optic nerve of a wild type mouse eye. (b) The corresponding STOC-T *en face* image and B-scan of the optic near head with superficial vessels, in a green and purple box, respectively. The scan locations and FOV are shown in the corresponding colors on the fundus image in panel (a). Scale bars represent 100  μm. (c) Two B-scans at the same location, with the focal plane set on two different layers, namely: on (or near) the photoreceptor layer and on the inner retina, for the blue and yellow box, respectively. (d) The corresponding color line graphs of the averaged central A-scans, normalized to the choroidal signal peak of the image in the blue box.

Figure 8 shows an example of the combined use of the fundus imaging system with the STOC-T system for volumetric mouse retinal imaging. Figure 8(a) displays the fundus image around the optic nerve of a wild-type mouse eye. The fundus camera FOV is useful for identifying features to guide the smaller STOC-T image FOV to areas of interest. The green box highlights the FOV for the STOC-T X and Y planes, and the purple line shows the location of the X and Z planes. The corresponding STOC-T *en face* image of the optic near the head with superficial vessels and B-scan is shown in Fig. 8(b).

Figure 8(c) presents two B-scans at the same location, with the focal plane set on two different layers. The first one, inside a blue box, shows the case where the focal plane is set on (or near) the photoreceptor layer, after following the focusing procedure driven by the calibrated fundus camera image. The second one, inside a yellow box, shows the case where the mouse position was shifted around 200 mm backward (away from the meniscus lens) compared with the previous one. Figure 8(d) shows the corresponding line graphs of the averaged central A-scans, normalized to the choroidal signal peak of the image in the blue box. As noticeable on a closer visual inspection of Fig. 8(c), and from Fig. 8(d), the SNR shift from the outer retina and choroid toward the inner retina and NFL is detectable although not as pronounced as it would be in flying-spot OCT images with the equivalent confocal gate imposed by a relatively large pupil. Although this more uniform SNR with depth complicates the real-time evaluation of whether the focal tuning procedure worked as intended, it gives a better chance for DAC to work at several depths, owing to a higher starting SNR away from the focus.

## 8 Appendix C: Custom Mouse Positioning System

**Fig. 9:**
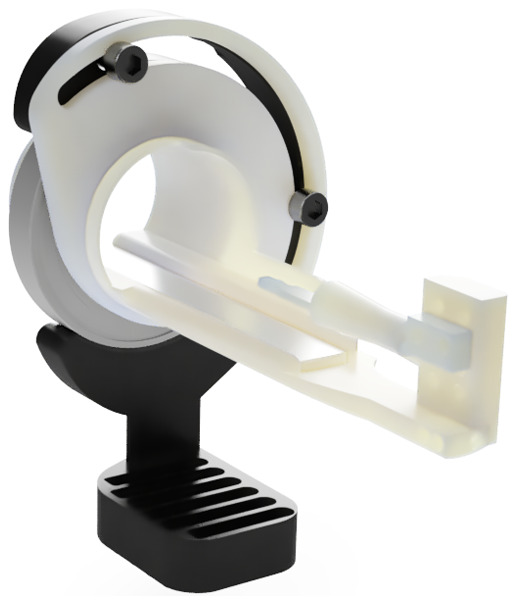
Dedicated mouse positioning system. The system incorporates an anesthesia delivery mechanism and a bite bar. In addition, it allows for the positional adjustment of the mouse along two degrees of freedom: a translational one aligned with the bite bar and the mouse’s spine, and a rotational one roughly about the mouse spine axis. This setup permits adjustments for a gimble configuration setting the pivot for the additional rotational degrees of freedom on or near the mouse right eye, using two additional rotational stages (not depicted). In turns, the setup was also placed on top of three motorized stages for the adjustment of the remaining translation degrees of freedoms along the *X*, *Y*, and *Z* axes.

One of the issues encountered during the course of the study was the proper alignment of the mouse eye with the optical axis of the STOC-T imaging system. To address this, a customized mouse positioning system was developed. The design of this system is illustrated in Fig. 9. The optomechanical stage we used could be translated along the X,Y, and Z axes with motorized stages and had a gimbal-like mount to house the mouse body, which placed the pivot for the rotational degrees of freedom on or near the mouse right eye when the mouse teeth were biting a bar incorporated in a custom-designed 3D-printed nozzle connected to the tube to the anesthesia apparatus. This system facilitated consistent and repeatable positioning of the mouse, significantly speeding up the setup process.

## 9 Appendix D: Supplementary Videos

Video 1. Time sequence of STOC-T en face amplitude images of an albino mouse chorio-retinal complex, averaged through the depth-ranges indicated in the sliced volume cross-sections in Fig. 4(d). On the right, the same three layers are shown as a time sequence of the averaged en face images following Gaussian filtering, amplitude squaring, and temporal mean removal (MP4; 43810.215 kb [URL: https://doi.org/10.1117/1.NPh.11.4.045003.s1]).

Video 2. Effect of mouse retinal blood flow on STOC-T signal in depth. Time sequence of the same dataset as in Video 1, for the NFL and choroidal layers and also for a B-scan cutting through a superficial vessel just below the NFL (MP4; 26689.648 kb [URL: https://doi.org/10.1117/1.NPh.11.4.045003.s2]).

Video 3. Spatially-resolved hemodynamics and biomechanics analysis performed with STOC-T on an albino mouse retina. Time sequence of both STOC-T en face amplitude images of the NFL of the mouse retina presented in Figs. 5 and 6 and an overlay of the corresponding mean-subtracted time sequence with the tissue displacement map (MP4; 16449.633 kb [URL: https://doi.org/10.1117/1.NPh.11.4.045003.s3]).

## Supplementary Material







## Data Availability

Data underlying the results presented in this paper are not publicly available at this time but may be obtained from the corresponding author upon reasonable request.
